# Breastfeeding in low-resource settings: Not a “small matter”

**DOI:** 10.1371/journal.pmed.1002646

**Published:** 2018-08-28

**Authors:** Lars Åke Persson

**Affiliations:** 1 Department of Disease Control, London School of Hygiene and Tropical Medicine, London, United Kingdom; 2 Ethiopian Public Health Institute, Addis Ababa, Ethiopia

## Abstract

Despite its clear biological benefits, many infants globally do not receive exclusive breastfeeding. In a Guest Editorial, Lars Åke Persson discusses what is needed to make breastfeeding the social norm.

My mother taught me the way of Acoli, and nobody should shout at me because I know the customs of our people.

When the baby cries let him suck milk from the breast.

There is no fixed time for breastfeeding.

When the baby cries it may be he is ill; the first medicine for a child is the breast.

While the medicine man is being called from the beer party.

From Okot P’Bitek: Song of Lawino [1]

Breastfeeding is the biological norm. Never before has the evidence base for optimal breastfeeding practices and their positive health effects been so robust [2]. Children who are breastfed for more extended periods have lower morbidity and mortality, and they benefit in neurocognitive function [2,3]. Evidence is emerging that recommended feeding patterns (i.e., breastfeeding within the first hour, exclusive breastfeeding for 6 months, and continued breastfeeding during the second year of life) may also reduce the long-term risk of overweight and diabetes [2]. A full-scale implementation of breastfeeding recommendations might prevent more than 800,000 annual child deaths before the age of 5 years [2]. Breastfeeding also confers several short- and long-term health benefits for the mother, including reduced risk of cancer [4].

Epidemiologist Cesar Victora has labelled breastfeeding a “biological dialogue” and breast milk a personalized medicine for infants [2,5]. The child conveys information to the mother about her needs by the frequency and intensity of suckling. The mother responds by altering the quantity and composition of her milk. The mother’s bacteria in the milk contribute to the child’s microbiota [6]. Breastfeeding protects against diarrhoea, pneumonia, and otitis media in younger children [2]. Therefore, the benefits of this mother–child biological communication go far beyond nutrition.

In spite of the established benefits for short- and long-term health, breastfeeding is not the global social norm. Adherence to the feeding recommendations is higher in low-income than in high-income societies, but the present level of adherence in impoverished settings still represents a significant missed opportunity in public health. Early initiation of breastfeeding is not the norm in sub-Saharan Africa; around half of neonates are breastfed within the first hour of life [7]. Only slightly more than one-third of children younger than 6 months in low- and middle-income countries are exclusively breastfed, and two-thirds of mothers in these settings adhere to the recommended continued breastfeeding during the second year of life [2].

What is needed for breastfeeding to become the social norm? The answers are complex but can be summarized as an enabling environment—at the societal level, within the health system, at the workplace, and in families [8]. At this point in history, evidence-based practices are available to create a vastly more enabling environment in low-resource settings, including in Africa, yet health system investments in breastfeeding have flagged [8], and national priorities focus elsewhere.

Only 1 out of 10 children worldwide are born in health institutions that can be labelled “baby friendly” [9]. In response, WHO and UNICEF have provided new implementation guidance to health systems for the support of breastfeeding. These guidelines emphasize the Baby-Friendly Hospital Initiative that has been around for a quarter of a century, and the Ten Steps for successful breastfeeding have been updated [9]. These steps of the Baby-Friendly Initiative try to secure a breastfeeding-enabling environment in the hospital with skilled and motivated health workers who support the mother in skin-to-skin contact with the newborn, early initiation, and continued breastfeeding. The evidence base for these steps is solid. Cochrane reviews have summarised the effectiveness of these interventions in maternity facilities for promoting early initiation of breastfeeding [10], as well as the accumulated evidence for the 6-month exclusive breastfeeding advice [11].

The family may be a breastfeeding-enabling environment, but members may also have a negative influence on breastfeeding. A qualitative study from South Africa found that fathers, grandmothers, or mothers-in-law may be significant others with an influential role in the mother’s decision-making regarding breastfeeding and other infant-feeding options [12]. Woman-to-woman or peer breastfeeding support has been shown to be an effective way of enabling breastfeeding at the individual or family level in a series of studies and systematic reviews [13]. These support opportunities should be leveraged and taken to scale.

At the societal level, governments are so intent on pursuing their economic goals that they neglect the importance of breastfeeding. In particular, the growing breastmilk substitute industry has a negative influence on breastfeeding with global losses in health, development, and survival [8]. In low-income settings, where the burden of infectious diseases is high, there are very few children for whom feeding with breastmilk substitutes is the best choice. There is clear global evidence of negative effects on recommended breastfeeding practices when the breastmilk substitute companies market their products with free samples in maternity facilities, through health workers, or in the media [14]. This clash between public health and commercial interests was in sharp relief at the recent World Health Assembly, where United States delegates sought to dilute a seemingly uncontroversial breastfeeding resolution in a way that would have protected the interests of formula companies [15]. After that, US government representatives reportedly tried to force countries to comply with this line by threats related to trade and military assistance. An anonymous voice from the Ecuadorian government expressed astonishment that a “small matter like breastfeeding” could elicit such a dramatic response.

Relatedly, in a recent article in *PLOS Medicine*, Pepita Barlow and colleagues reported a document analysis of trade challenges at the World Trade Organization concerning national noncommunicable disease prevention policies [16]. When designing regulations to promote public health, such as labelling requirements and food-quality standards, policymakers in low-income countries appeared to face significant negative pressure from large country partners with whom they had trade agreements. When considering economic growth, governing officials should be aware that, in addition to the costs of non-breastfeeding to health and well-being, cognitive deficits associated with present levels of non-breastfeeding have been estimated to underlie annual economic losses of around $302 billion globally [8]. There are also health system costs related to the morbidity and mortality caused by non-breastfeeding. In addition, the environmental costs of breastmilk substitutes are high [8].

Is investment in breastfeeding relevant to the global commitments made in the Sustainable Development Goals? There is no doubt that optimizing breastfeeding practices is central for the third goal because of its significant effects on maternal health, child health, and chronic disease risk. Breastfeeding is clearly essential for the second goal (nutrition). Furthermore, improved breastfeeding could contribute to the fourth goal (education) due to its positive effects on cognitive function and to the tenth goal by reducing inequalities [2]. Now is the time to take the substantial evidence for the efficacy of breastfeeding promotion interventions seriously and move into the implementation research and scale-up stage. Investments and reinforced political support are needed to enable breastfeeding to become the global social norm.
